# Conference on Emerging Foodborne Pathogens

**DOI:** 10.3201/eid0204.960423

**Published:** 1996

**Authors:** Diane Dalisera

**Affiliations:** Emerging Foodborne Pathogens Conference International Life Sciences Institute (ILSI), Washington, DC, USA


222. Vogel P, Abplanalp D, Kell W, et al. Venezuelan equine encephalitis in BALB/c mice: kinetic analysis of central nervous system infection following aerosol or subcutaneous inoculation. Arch Pathol Lab Med 1996;120:164-72.
223. Waiyaki PG. Cholera: its story in Africa with special reference to Kenya and other east African countries. East Afr Med J 1996;73:40-3.
224. Walker DH, Barbour AG, Oliver JH, et al. Emerging bacterial zoonotic and vector-borne diseases: ecologic and epidemiologic factors. JAMA 1996;275:463-9.
225. Wallace MR, Sharp TW, Smoak B, et al. Malaria among US troops in Somalia. Am J Med 1996;100:49-55.
226. Wann S-R, Liu Y-C, Yen M-Y, Wang J-S, Chen Y-S, Wang J-H, Cheng D-L. Endogenous *Escherichia coli* endophthalmitis. J Formos Med Assoc 1996;95:56-60.
227. Warren R, Hauman J, Beyers N, et al. Unexpectedly high strain diversity of *M tuberculosis* in a high incidence community. S Afr Med J 1996;86:45-9.
228. Weissenbacher M, Cura E, Segura E, et al. Serological evidence of human hantavirus infection in Argentina, Bolivia and Uruguay. Medicina 1996;56:17-22.
229. Whitcup SM. Ocular manifestations of AIDS. JAMA 1996;275:142-4.
230. Wilkinson D, de Cock KM. Tuberculosis control in South Africa - time for a new paradigm? S Afr Med J 1996;86:33-5.
231. Wilkinson D, Moore DAJ. HIV-related tuberculosis in South Africa - clinical features and outcome. S Afr Med J. 1996;86:64-7.
232. Williamson H. Treatment of acute bronchitis: there's much work to be done. Arch Fam Med 1996;5
233. Winker MA, Flanagin A. Infectious diseases: a global approach to a global problem. JAMA 1996;275:245-6.
234. Woelffer GB, Bradford WZ, Paz A, Small PM. A computer assisted molecular epidemiologic approach for confronting the reemergences of tuberculosis. Am J Med Sci 1996;311:17-22.
235. Xu J, Cheng B, Wu Y, et al. A new diarrhea pathogen: entero-adherent-invasive-toxigenic *Escherichia coli.* Chin Med J 1996;109:16-7.
236. Yahav J, Oderda G, Diver-Haber A, et al. Serum pepsinogen I in childhood *Helicobacter pylori* gastritis: its relation to mucosal peptic activity. Isr J Med Sc 1996;32:56-9.
237. Yao C, Wang W-W, Chung Y-M, Su Y-L, Liu C-Y, Chen Y-M A. Transfusion-transmitted AIDS: report of the first case in Taiwan. J Formos Med Assoc 1996;95:51-5.
238. Young LHY. Therapy for cytomegalovirus retinitis: still no silver lining. JAMA 1996;275:149-50.
239. Ytterdahl T. Tropical diseases: an interview with David Stevenson, MD. Tidsskr Nor Laegeforen 1996;116:(January):pages not yet available.
240. Zaki SR, Khan AS, Goodman, et al. Retrospective diagnosis of hantavirus pulmonary syndrome, 1978-1993: implications for emerging infectious diseases. Arch Pathol Lab Med 1996;120:134-9.
241. Zhu CM. Comments on some new viruses associated with old diseases in China. Chin Med J 1996;109:5-10.

### Conference on Emerging Foodborne Pathogens

The conference on "Emerging Foodborne Pathogens: Implications and Control," March 24-26, 1997, Radisson Plaza Hotel at Mark Center, Alexandria, Virginia, USA, is organized by the International Life Sciences Institute (ILSI), ILSI North America Technical Committee on Food Microbiology, the U.S. Centers for Disease Control and Prevention, Department of Agriculture, and Food and Drug Administration, in cooperation with the Food and Agriculture Organization of the United Nations, and the Pan American Health Organization/World Health Organization. The conference will review the lessons learned and knowledge gained concerning the emergence/reemergence and dissemination of food-related microbial threats to health; identify factors that foster emergence/reemergence and dissemination of these hazards; identify scientific and food safety strategies to address emerging foodborne microbial hazards; and identify future research needs. The conference will be of special interest to food protection and public health professionals, including microbiologists, epidemiologists, physicians, and health policy makers; industry, academic, and government researchers; and others interested in microbial food safety hazards.

For program and registration information, contact

Ms. Diane Dalisera
Emerging Foodborne Pathogens Conference
International Life Sciences Institute (ILSI)
1126 Sixteenth Street, NW
Washington, DC 20036-4810, USA
Telephone: 202-659-0074
Fax: 202-659-3859
E-mail: meetings@dc.ilsi.org

The conference on "Emerging Foodborne Pathogens: Implications and Control," March 24-26, 1997, Radisson Plaza Hotel at Mark Center, Alexandria, Virginia, USA, is organized by the International Life Sciences Institute (ILSI), ILSI North America Technical Committee on Food Microbiology, the U.S. Centers for Disease Control and Prevention, Department of Agriculture, and Food and Drug Administration, in cooperation with the Food and Agriculture Organization of the United Nations, and the Pan American Health Organization/World Health Organization. The conference will review the lessons learned and knowledge gained concerning the emergence/reemergence and dissemination of food-related microbial threats to health; identify factors that foster emergence/reemergence and dissemination of these hazards; identify scientific and food safety strategies to address emerging foodborne microbial hazards; and identify future research needs. The conference will be of special interest to food protection and public health professionals, including microbiologists, epidemiologists, physicians, and health policy makers; industry, academic, and government researchers; and others interested in microbial food safety hazards.

For program and registration information, contact
